# Supplementary material for: Treatment success rate and associated factors among drug susceptible tuberculosis individuals in St. Kizito Hospital, Matany, Napak district, Karamoja region. A retrospective study
Source: PLoS One. 2024 May 14;19(5):e0300916. doi: 10.1371/journal.pone.0300916 (PMC11093355; doi:10.1371/journal.pone.0300916)
Supplement: Attachment — Submitted filename: Review comments.docx [file pone.0300916.s002.docx]

1. Inclusion and exclusion: I am afraid that excluding those with missing age, gender and disease type would introduce bias in the estimates. The authors need to assess if complete case analysis did not produce biased results. By doing multiple imputation and then compare results (complete case vs imputed data) to check if there is consistence in the findings would help.

2. Inclusion and exclusion: Since those not evaluated were also initiated on treatment, I think they should be part of the denominator. Excluding them especially if they are many can lead to overestimation of the treatment success rate. Can the authors estimate the treatment success rate with the "not evaluated" included?

3. Inclusion and exclusion: Please clarify why the multi-drug resistant TB patients were excluded.

4. Under methods and materials, the authors mention to have used a customized proforma to abstract data. A reference to this proforma at its first mention would be helpful.

5. Study variables: A clear definition of the primary outcome should be given. I suppose this was a proportion. If yes, please define the primary outcome mentioning the numerator and the denominator.

6. Under data management and analysis, the authors mention to have used a modified poison regression to estimate prevalence ratios. Did the authors fit the model with robust standard errors? Since Poison regression if used for binary outcome, it can estimate confidence interval for the prevalence that exceeds a 1, and consequently this affects estimates for the prevalence ratio. Please check that robust standard errors were used and mention this in the analysis methods.

7. It's not clear how a multivariable Poison regression model was built. What factors were considered in the multivariable model and how did the authors arrive at a final fit. Also, how did they decide which factors to include in the model? All these details are needed to make things clear and for reproducibility reasons.

8. Under results in Table 2, it seems like all variables in the data were included in the multivariable analysis and no model building was considered. Would the results be different if only variables with say a p-value at bivariable analysis less than 0.1 were included? Did the authors check for multicollinearity? What about overdispersion, noting that Poisson model assumes equality of variance and mean, which assumption if it does not hold, the model can give misleading results. A negative binomial would be helpful.

9. In reference to my point 1, would the results in Table 2 remain the same if missing data were considered (instead of excluding them)?

10. Below table 2, statements like "Nutrition status", "HIV status" and others look like hanging statements. Please revise the paragraph as you list the significant factors.

11. Please add line numbers to the manuscript as this will make the review process easier.

12. Somewhere you are missing full stops. For example, a full stop is missing before the statement that begins "We used chis-square to test ..."
